# Orexitropic Signaling Proteins in Obese Children

**DOI:** 10.1100/tsw.2007.218

**Published:** 2007-08-24

**Authors:** Ljiljana Saranac, Bojko Bjelakovic, Hristina Stamenkovic, Borislav Kamenov

**Affiliations:** Pediatric Clinic, University Clinical Centre, Nis, Serbia

**Keywords:** leptin, NPY, obesity, orexitropic signaling

## Abstract

Adipose tissue is not only the main organ for energy storage, but it also has endocrine properties, producing “adipokines” responsible for energy homeostasis, insulin sensitivity, and inflammation. Leptin, produced by adipocytes, is the key hormone in appetite regulation and suppression of orexigenic, hypothalamic neuropeptide Y (NPY). We wanted to establish and compare levels of leptin and NPY in different obesity types in childhood, and to investigate their correlations with auxological parameters. Twenty-one obese children (seven girls and 14 boys), divided into two groups, were compared with 14 controls. The mean age of the study group was 10.81 ± 3.69 years and the mean puberty stage was 2.21. The mean body mass index (BMI) was 32.80 kg/m (range 23.30– 47.02) and the mean overweight 30.73 kg (range 8.00–74.00). The mean leptin level was higher in boys and in the group with central obesity, but was not significant. Leptin/NPY ratio and leptin/BMI ratio was also higher in the central obesity group and there was a more significant difference compared with controls. We found significant correlation of the leptin level with body mass (BM), body mass excess (BME), and BMI (p < 0.05). The mean leptin level in obese children was very high (36.39 ng/ml). Leptin and NPY levels showed inverse values in two different obesity types. Results are suggestive for leptin resistance rather than leptin deficiency in our group of obese children. Orexitropic signaling proteins correlated significantly with auxological parameters. Determination of the leptin and NPY concentrations provided evidence that obesity represents disease with neuroendocrine dysfunction and high leptin/NPY ratio, which could be a useful marker for central obesity.

### INTRODUCTION

Obesity in children is a problem of major scientific, health-related, and economic significance, which today is reaching pandemic proportions. In comparison with the large literature on adults, obesity studies in children are still comparably few.

Since the discovery of leptin in 1994, it has been assumed that adipose tissue is not just a fat storage organ, but plays an important role in many physiological and pathological processes, including appetite regulation, glucose homeostasis, immune response, growth and differentiation, angiogenesis, hypertension, atherosclerosis, and cancer[1,2,3]. Mature adipocytes have the potential to secrete leptin and adiponectin, resistin, ASP (acylation-stimulating protein), TNF-alpha (tumor necrosis factor-alpha), PAI-1 (plasminogen activator inhibitor-1), interleukin-6, and other biologically active molecules. Leptin is a gene product almost exclusively produced by adipocytes. The name is derived from the Greek word “leptos”, which means “thin”. This complex, pleiotropic adipocytokine is capable of both favorable and adverse effects. Among the first actions discovered in rats and confirmed in humans was that it signals to the brain that the stomach is full and suppresses production of NPY (neuropeptide Y), a stimulator of food intake[3,4,5,6]. It controls the start of puberty[7]; stimulates sympathetic nervous system activity and energy expenditure; influences thyroid, growth, and sex hormone axes[2,3,4,5,6,7]; acts as a growth hormone stimulator; plays a role in respiration control[8]; inhibits the release of CRH (stress response control)[9,10]; influences ovulation and sex hormone level (reproduction control)[11,12,13,14,15]; stimulates phagocytosis and Th1 cytokine production (immune response control)[16]; causes insulin resistance in high concentrations[17]; exaggerates oxidative stress of endothelial cells and vascular calcifying effect; and influences body mass density[18]. Leptin receptors are expressed in the hypothalamus, chorioid plexus, but also in gonads, adipose tissue, and hemopoietic organs[2].

NPY is synthetized in the hypothalamic arcuate nucleus and in the hypothalamic paraventricular nucleus. It is a powerful stimulator of food intake when applied centrally. It also influences the autonomic nervous system, favoring parasympathetic efferents. Human obesity may partly occur as a result of dysregulation of a central-peripheral loop system, involving the brain, hypothalamus, and adipose tissue. Leptin appears to be a crucial hormone in this system, which provides negative feedback on hypothalamic NPY and permits closure of the loop between the hypothalamus and the periphery, thereby maintaining a normal body homeostasis[1,2,3,4,19]. Knowledge about this interplay between orexitropic signals (orexigenic and appetite suppressors) may provide the keys to our understanding of human obesity.

The aim of the study was to establish and compare leptin and NPY serum levels in children with different obesity types, and to investigate their correlations with auxological data in boys and girls.

### METHODS

This study was designed as cross-sectional and patients were ambulatory. Informed consent was obtained from each patient and parents, while the Review Board of the Children's Hospital approved the study.

Obese children (seven girls and 14 boys) referred to the Pediatric Clinic for examination because of body mass excess were divided into two groups based on obesity type according to subcutaneous fat distribution and waist/hip ratio[20]: a central (12 patients) and peripheral obesity type (nine patients) group. The control group consisted of 14 children with ideal body weight for height; matched for age, sex, and pubertal stage. Auxological parameters (body height and body weight) were obtained and body mass index (BMI) ($kg/m^2$) calculated. Body height percentile and pubertal stage were determined by Tanner Whitehouse standards[21], and height standard deviations score for chronological age (SDSht) calculated. Height age was defined as the age at which the individual’s height was at the 50th percentile. Body mass excess (BME) was calculated in kilograms and percentages.

Blood samples were drawn in the morning after an overnight fast. Serum leptin concentrations were determined by a human leptin RIA method (DRG International Inc., USA). The limit of sensitivity for the leptin assay was 0.5 ng/ml. Intra- and interassay coefficients of variation (CV) were 4.6 and 5.0%, respectively. Serum NPY was also determined by the RIA method (DRG International Inc., USA). The intra- and interassay CV was 3.9 and 2.6%, respectively.

### Statistical Analysis

Data are presented as means ± SD. Comparisons between groups were calculated by one-way ANOVA. The relationships between variables were determined by linear regression analysis before and after log transformation of serum leptin concentrations in order to limit dispersion of the variance. A value of $p < 0.05$ was chosen as the level of significance. Statistical analyses were performed using SPSS 12.0 software for Windows.

## RESULTS

The mean age of the study group was 10.81 ± 3.69 years and the mean puberty stage was 2.21. Obese children had lipomasthia, genua valga, and boys (with only two exceptions) pseudohypogenitalismus. Mean puberty stage for boys was 1.89 and for girls 2.80. Obese girls showed earlier puberty (mean age for P2 puberty stage was 9 years) in comparison with the opposite sex, which entered P2 at average age of 11.2 years. The mean BMI was very high: 32.80 kg/m$^2$, ranging from 23.30 to 47.02. The mean height percentile was P69.71 (0.95 ± 1.21 SDSht) and documented that children had optimal growth. Only in female children with peripheral obesity did we find a significantly higher difference in SD of body height in comparison with controls ($p < 0.05$). Overweight was reaching abnormal values: mean overweight being 30.73 kg (ranging from 8 to 74) (Table 1).

**TABLE 1**
**Auxological Data (Mean ± SD, Range)**

| Variable | Boys (Study Group) | Girls (Study Group) | Study Group (Total) | Control Group | *p* |
|---|---|---|---|---|---|
| n | 14 | 7 | 21 | 14 | |
| Age (years) | 10.69 ± 3.20<br>5.00 – 15.08 | 11.06 ± 4.80<br>5.00 – 16.58 | 10.81 ± 3.69<br>5.00 – 16.58 | 10.79 ± 2.67<br>5.50 – 15.00 | ns [a]<br>ns [b] |
| Height age (years) | 15.99 ± 16.23<br>6.50 – 71.00 | 11.12 ± 4.23<br>5.75 – 18.50 | 14.36 ± 13.50<br>5.75 – 71.00 | 11.23 ± 3.09<br>6.00 – 16.00 | ns [a]<br>ns [b] |
| Ht (height in cm) | 146.58 ± 18.98<br>118.50 – 179.50 | 142.93 ± 21.29<br>112.00 – 173.00 | 145.36 ± 19.32<br>112.0 – 179.50 | 143.43 ± 16.13<br>115.00 – 164.00 | ns [a]<br>ns [b] |
| PHt (percentile of Ht) | 73.00 ± 27.50<br>25.00 – 95.00 | 63.14 ± 32.90<br>15.00 – 95.00 | 69.71 ± 28.96<br>15.00 – 95.00 | 57.79 ± 25.23<br>8.00 – 97.00 | ns [a]<br>ns [b] |
| SDSht (height SD score) | 1.07 ± 1.23<br>–1.00 – 2.49 | 0.71 ± 1.22<br>–0.80 – 2.10 | 0.95 ± 1.21<br>–1.00 – 2.49 | 0.34 ± 0.92<br>–1.40 – 1.84 | ns [a]<br>ns [b] |
| BM (kg) | 73.66 ± 26.34<br>44.00 – 132.0 | 64.14 ± 25.94<br>30.00 – 98.00 | 70.49 ± 25.96<br>30.00 – 132.00 | 37.49 ± 12.38<br>20.00 – 57.00 | ns [a]<br>*** [b] |
| BME (kg) | 33.19 ± 17.59<br>8.00 – 74.00 | 25.81 ± 17.65<br>9.70 – 54.00 | 30.73 ± 17.53<br>8.00 – 74.00 | –0.41 ± 1.90<br>–3.50 – 3.00 | ns [a]<br>*** [b] |
| BMI (kg/m$^2$) | 34.06 ± 6.38<br>25.72 – 47.02 | 30.28 ± 6.66<br>23.30 – 40.50 | 32.80 ± 6.56<br>23.30 – 47.02 | 17.62 ± 2.17<br>14.40 – 21.50 | ns [a]<br>*** [b] |
| Puberty stage | 1.89 ± 0.78<br>1.00 – 3.00 | 2.80 ± 1.64<br>1.00 – 5.00 | 2.21 ± 1.19<br>1.00 – 5.00 | 2.21 ± 1.25<br>1.00 – 5.00 | ns [a]<br>ns [b] |

[a] Boys vs. girls; [b] study group vs. control group; ns, no significance; *** $p < 0.001$.

The mean leptin level was higher in boys and in the group with central obesity, but not significant. Leptin/NPY ratio and leptin/BMI ratio was also higher in the central obesity group. NPY levels were higher in boys and in the group with peripheral obesity. The central obesity group had more prominent difference in leptin level, log of leptin level, NPY, leptin/NPY, and leptin/BMI ratio compared with controls (Table 2). Leptin level in the study group was very high (36.39 ng/ml). Leptin and NPY levels showed inverse values in two different obesity types (Figs. 1 and 2).

**TABLE 2**
**Orexitropic Signaling Proteins in Investigated Children (Mean ± SD, Range)**

| Variable | Study Group | Boys | Girls | Central Obesity | Peripheral Obesity | Control Group |
|---|---|---|---|---|---|---|
| n | 21 | 14 | 7 | 12 | 9 | 14 |
| Leptin (ng/ml) | 36.39 ± 43.25<br>6.20 – 174.00 | 37.46 ± 49.41<br>6.20 – 174.00 | 34.24 ± 28.77<br>7.20 – 69.00 | 49.93 ± 51.72 [b**]<br>6.20 – 174.00 | 18.34 ± 18.89 [c*]<br>6.20 – 56.00 | 4.78 ± 2.51 [a*]<br>2.00 – 11.00 |
| Log leptin | 1.30 ± 0.47<br>0.79 – 2.24 | 1.28 ± 0.50<br>0.79 – 2.24 | 1.36 ± 0.44<br>0.86 – 1.84 | 1.46 ± 0.50 [b***]<br>0.79 – 2.24 | 1.10 ± 0.37 [c***]<br>0.79 – 1.75 | 0.63 ± 0.21 [a***]<br>0.30 – 1.04 |
| NPY (ngl/ml) | 1.65 ± 2.48<br>0.11 – 8.80 | 2.09 ± 2.95<br>0.11 – 8.80 | 0.79 ± 0.55<br>0.11 – 1.50 | 1.23 ± 1.50 [b*]<br>0.11 – 5.20 | 2.22 ± 3.42<br>0.11 – 8.80 | 2.95 ± 2.00<br>0.80 – 6.10 |
| Leptin/NPY | 50.13 ± 44.67<br>0.81 – 181.82 | 46.21 ± 38.56<br>0.81 – 118.16 | 57.98 ± 57.62<br>7.74 – 181.82 | 52.34 ± 26.75 [b***]<br>12.00 – 103.64 | 47.19 ± 63.14 [c*]<br>0.81 – 181.82 | 2.11 ± 1.18 [a***]<br>0.80 – 4.63 |
| Leptin/BMI | 1.03 ± 1.06<br>0.19 – 3.70 | 1.01 ± 1.18<br>0.19 – 3.70 | 1.09 ± 0.88<br>0.26 – 2.14 | 1.32 ± 1.22 [b**]<br>0.19 – 3.70 | 0.65 ± 0.71<br>0.20 – 2.14 | 0.27 ± 0.14 [a*]<br>0.13 – 0.66 |

[a] Study group vs. control group; [b] central obesity group vs. control group; [c] peripheral obesity group vs. control group; * $p < 0.05$; ** $p < 0.01$; *** $p < 0.001$.

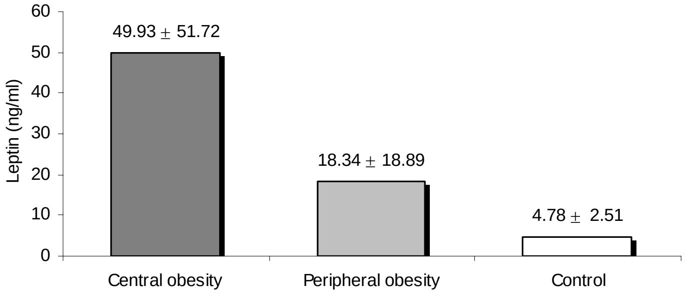

**FIGURE 1.** Leptin levels in different obesity types and control (mean ± SD).

We found significant correlation of the leptin level with BM, BME, and BMI ($p < 0.05$) (Fig. 3).

### DISCUSSION

Modern studies of obesity strongly indicate that this is a multifactorial problem, which could be a consequence of dysregulation of endocrine function of adipose tissue and its communication with the

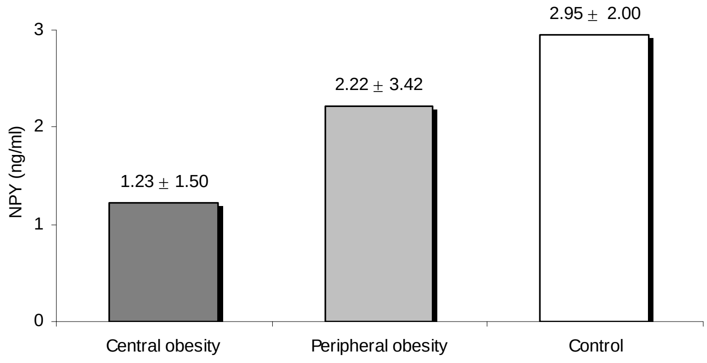

**FIGURE 2**. NPY levels in different obesity types and control (mean ± SD).

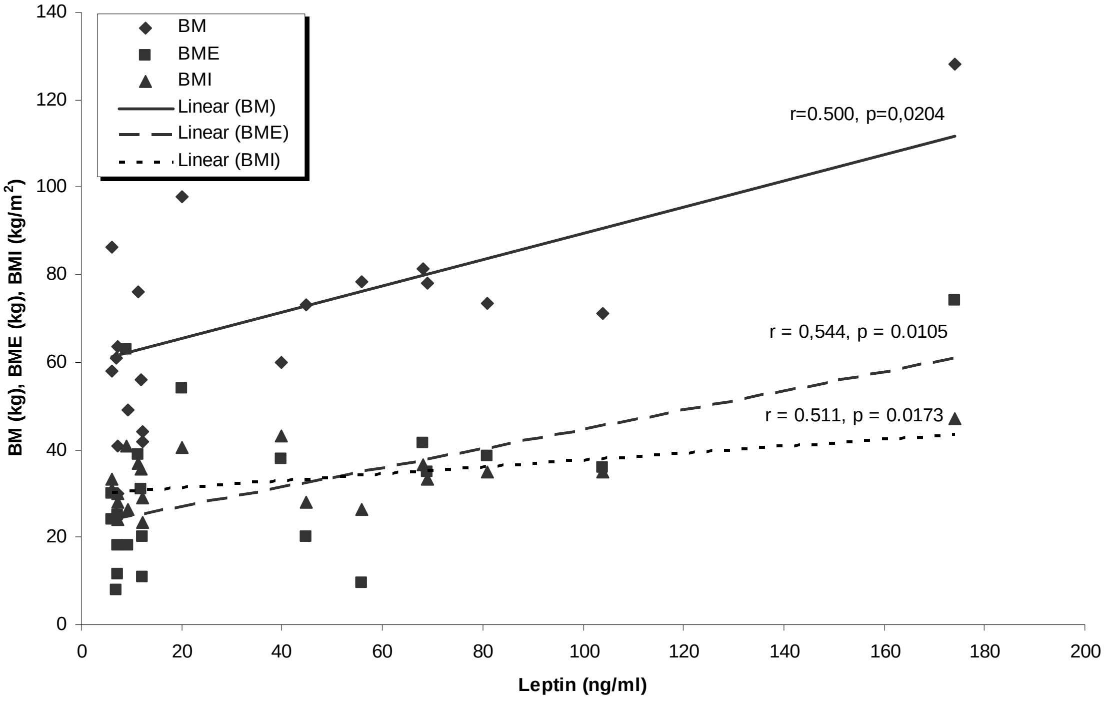

**FIGURE 3**. Correlations of leptin levels with BM, BME, and BMI.

hypothalamus. It has always been a mystery how total body fat could communicate with the brain. Now it is clearly recognized that fat communicates with the brain via leptin[22,23]. The status of body energy stores has critical survival value and, therefore, has been the object of potent selection pressure in evolution. Leptin is a plausible candidate for humoral signal acting in an endocrine fashion by reporting the size of adipose tissue mass to the hypothalamic center. So it would deserve to be named "satiety protein", a molecule integrating somatic energy stores, energy expenditure, and fertility, or a biological radar system for the assessment of body mass and metabolic gate for achieving puberty[1,2,3,4,5,6,7,23].

Plasma leptin concentrations correlate with the amount of energy stored as fat, and obese individuals express higher levels of leptin than lean individuals[2,5,22]. Our study confirmed this positive correlation between leptin concentrations and BM, BMI, and BME. Height of investigated children was optimal (mean percentile being P69.71, +0.95 SD). High caloric and protein intakes in overfed children provide full energy stores, high leptin values, and stimulation of growth hormone secretion and T4 to T3 conversion. This could explain the excellent growth in our study group. Growth hormone (GH) stimulates this hormonal conversion and this effect is more prominent in children with higher GH sensitivity and growth velocity[24]. In addition, leptin per se stimulates T4 to T3 conversion and leptin receptors are identified in the thyroid gland[25]. Leptin, GH, and thyroid hormones, acting synergistically, may be responsible for stimulation of growth in children with exogenous obesity.

The mean puberty stage in our study group was 2.21, which is advanced for mean chronological age. In obese girls, we noticed earlier puberty start, opposite to obese boys, who presented with sexual infantilism, pseudohypogenitalismus, and lipomasthia. A prepubertal rise in serum leptin concentration has been proposed as a “trigger” for the onset of puberty in boys[7]. The leptin story has restored credibility to the critical weight hypothesis originally proposed by Rose Frish in the 1970s. The critical weight hypothesis states that the onset and regularity of menstrual function necessitates maintaining weight above a critical level and, therefore, above a critical amount of body fat. Amenorrhea and infertility are observed frequently in individuals who maintain a reduced body weight through vigorous exercise and/or caloric restriction[11,12,13,14]. A signaling system whereby energy is sensed by physiological systems integrating energy homeostasis (feeding behavior and energy expenditure), somatic growth, and fertility has critical survival value[7]. It is commonly thought that the onset of puberty requires good nutritional condition. In normal girls, serum leptin increases before the rise of reproductive hormones related to puberty. In juvenile female mice, injection of leptin induces earlier maturation of the reproductive system. In the study of Tezuka et al., leptin stimulated the secretion of gonadotropin in juvenile rats. These findings suggested that leptin plays a physiological role in the juvenile period[26]. Leptin acts directly on pituitary cells (even in the juvenile period), stimulates the secretion of gonadotropins, and modulates the response of the pituitary in the period before puberty. Excessive leptin concentration also interferes with reproductive function. Hyperleptinemia caused by extreme obesity may disrupt pituitary function and impair gonadotropin secretion during the peri- and postpubertal period in humans. These data could explain sexual dimorphism in pubertal development in investigated obese children.

We did not discover any case of leptin deficiency. Despite results in rodents, human obesity is rarely the result of inherited leptin deficiency (ob gene mutation). There are few studies demonstrating this defect in Pakistani and Turkish children successfully treated by recombinant leptin[27,28]. More often, there is leptin resistance in human obesity. The mechanisms underlying leptin resistance may include abnormalities of leptin secretion, defective transport of leptin into the brain, and/or reduced hypothalamic leptin signaling. The pathogenesis of leptin resistance is currently under intense investigation. Our group of obese children had very high mean leptin value, in accordance with this statement. Hyperleptinemia, especially high leptin levels found in the central obesity group with unsuppressed NPY, in both types of obesity is, in our opinion, a reflection of leptin resistance. Unexpectedly, we found higher, but not significant, values in boys, while taking into account that female subjects have higher percentages of body fat and proportionally higher leptin values. The majority of the studies with a gender difference were based on simple measurement of body composition, such as BMI, bioimpedance, anthropometric index of abdominal fat distribution, and/or the waist-hip ratio. However, when robust body composition techniques, Dual Energy X-ray (DEXA), and diet control measures (isoenergetic levels) were taken into consideration, the relationship between log plasma leptin concentrations and body fat percentage was not different in men and women[25].

The increase in incidence of type 2 diabetes now observed in adolescence is a “first consequence” of the epidemic of obesity among young people[29,30,31]. Fat distribution and, specifically, visceral (central) fat accumulation, appears to be a strong risk predictor for cardiovascular disease (CVD). The term “the Deadly Quartet” was used to define the association of central obesity, glucose intolerance, hypertriglyceridemia, and hypertension[19]. Most recent studies confirm that fat distribution is a better predictor of risk factors for CVD than body weight or BMI. Hyperuricemia and polycythemia have been found to be significantly more prevalent in subjects with visceral obesity, as well as elevated C-reactive protein, hyperhomocystinemia, microalbuminuria, and PAI-I[32,33]. Insulin resistance and a state of chronic inflammation are also underlying factors contributing to this heterogeneous disorder, named metabolic syndrome. Every year, a new laboratory variable is added to the cluster of already-established criteria of metabolic syndrome. As Edwin Gale noticed, the answer to this question has more heads than a hydra[34]. In childhood, one-third of the metabolic syndrome is undiagnosed and the mentioned laboratory data remain within normal limit for a long period. We noticed cases of extreme clinical obesity with 100% overweight, genua valga, lipomasthia, and ideal blood glucose, lipids, slightly elevated CRP and C peptide. Only leptin level was seriously increased, in some cases tripled or severalfold higher than the upper limit of the normal value. Then, finally, parents and their obese children realized that the child was not "obese and healthy" and started dieting. We found elevated leptin/NPY ratio in the central obesity group and inverse values of leptin and NPY in the central and peripheral obesity type. So, this disturbed interplay between orexitropic signals could be a useful marker for central obesity, but further studies in larger cohorts are necessary. Central obesity as a critical sign of metabolic syndrome may also have disturbed adipokine levels. Matsubara et al.[35] have found low levels of adiponectin, the other most abundant adipokine (hypoadiponectinemia) in central obesity. Leptin correlated with insulin resistance. Recent data suggested that adipose tissue might contribute to the inflammation, because both leptin and adiponectin may play a role in the development of metabolic syndrome, so hyperleptinemia and hypoadiponectinemia can be considered risk factors for CVD. Hyperleptinemia as equivalent of leptin resistance or impaired balance between main adipokines could be the unique detected metabolic abnormality in obese subjects.

Hyperleptinemia and unsuppressed orexigenic NPY in our study demonstrated resistance to leptin as appetite suppressor in obese children. Leptin resistance was higher with greater significance in central than in peripheral obesity (Table 2). The higher the leptin/NPY ratio, the greater the leptin resistance. Impaired balance between orexitropic signals as an early biological marker of the central obesity in children found in our study deserves further examination. Serum NPY concentration as reflection of central NPY concentration is feasible to measure. The neurons that responded to NPY originated in the arcuate nucleus and projects into the paraventricular and dorsomedial nuclei. The arcuate nucleus lies outside the blood-brain barrier (there is no blood-brain barrier in the medial basal hypothalamus) and can be reached by leptin in the circulation[23].

Although the leptin mechanism offers the potential for new treatments for obesity, it is not easy to achieve. Administration of exogenous leptin may overcome resistance to hormone, but the timing is very important (postobese state, SGA babies, nocturnal application). The postobese state is characterized by adipose tissue hyperplasia and lower leptin production, or a relative hypoleptinemia[36]. The recent finding that leptin can modulate both synapse numbers and synaptic activity in NPY and POMC neurons in the hypothalamic arcuate nucleus are consistent with the concept that under- and overnutrition during critical periods of high hypothalamic development may induce long-lasting and potentially irreversible effects into adulthood[37]. A small subpopulation of leptin-deficient subjects will benefit most from leptin supplementation[27,28].

### CONCLUSIONS

Our results are suggestive for leptin resistance rather than leptin deficiency in investigated children. Leptin and NPY levels showed inverse values in central and peripheral obesity. Hyperleptinemia and unsuppressed orexigenic NPY in our study were more prominent in central obesity and documented resistance to leptin as appetite suppressor in obese children. Determination of orexitropic signals provides evidence that obesity represents disease with neuroendocrine dysfunction and high leptin/NPY ratio could be a useful marker for central obesity. This increasing knowledge of endocrine properties of adipose tissue shines new light in a very dark box of brain mechanisms responsible for obesity. New therapeutic approaches targeting leptin brought a lot of enthusiasm for treatment of obesity, especially in this sensitive period of life.
